# Obituary for Dr. Konstantin Danilenko (19.03.1962–18.01.2023)

**DOI:** 10.3390/clockssleep5010010

**Published:** 2023-03-02

**Authors:** Arcady A. Putilov

**Affiliations:** 1 Research Group for Math-Modeling of Biomedical Systems, Research Institute for Molecular Biology and Biophysics of the Federal Research Centre for Fundamental and Translational Medicine, 630117 Novosibirsk, Russia; putilov@ngs.ru; 2 Laboratory of Nanobiotechnology and Biophysics, North-Caucasus Federal University, 355029 Stavropol, Russia; 3 Laboratory of Sleep/Wake Neurobiology, Institute of Higher Nervous Activity and Neurophysiology of the Russian Academy of Sciences, 117865 Moscow, Russia

Dr. Konstantin V. Danilenko (Figure 1), just 60 years old, died on 21 January 2023 of cancer. We are all deeply saddened and devastated by this completely unexpected loss [1,2].

After graduating from the Novosibirsk Medical University, he joined my laboratory in 1988 as a PhD student. We had just started to work at the large medical research complex located in Elzovka near Novosibirsk, which included several research institutes and hospitals. Here, in Elzovka, he met his future wife, Elena, at a New Year’s party. Family was important to him. Konstantin was a loving husband and parent. He is survived by his wife and daughter, Nataliya, who also became a scientist.

Affectionately known as Kostya [1], he cooperated with many other colleagues from my laboratory to study the beneficial effects of bright light treatment (BLT) for seasonal affective disorder (SAD), for which he had an incredible passion. His PhD term took place in both the Perestroika and post-Perestroika periods when, in the USSR, BLT for SAD was nothing but an exotic intervention for an unknown disorder [3]. During these periods, biomedical studies embraced various challenges and adversities. The conditions for his research were unique in many respects. On the one hand, we managed to offer to our study volunteers pretreatment, treatment, and posttreatment days at the hospital. We even offered them one week during winter at the southernmost resort of the former Soviet Union near Ashgabat (now Turkmenistan) to assess the therapeutic effects of this natural treatment. On the other hand, we were unable to find kits and equipment for some of biochemical analyses anywhere in the country [4]. Therefore, Kostya’s visits to the so-called “capitalist countries” started with a trip Novosibirsk-Magadan-Anchorage-Fairbanks to assay melatonin in blood samples collected in Siberia at the University of Alaska Fairbanks [5]. He also used to travel to Chukotka, the region on the opposite side of Bering Strait, to collect questionnaire data on SAD prevalence in small villages populated by Chukchee, Inuit, and eastern Slavic peoples.

Kostya was talented and hard worker, a person who showed exemplary effort, was honest with strong moral principles, and a man of integrity. He was known to everyone in the laboratory for being energetic, diligent, organized, and conscientious. Kostya made our work on this BLT research very efficacious and pleasurable. I can only assume that such biomedical research was in his blood. He became its heart and soul, and, in general, he offered a level of optimism that was honest, heartfelt, and spilled over into all that he did in the lab. His pleasant personality was contagious to everyone he worked with, everyone felt his big heart and giving nature and was touched by his kindness, politeness, and mild temperament. He was always a peacemaker and a source of positivity, had a remarkable ability to connect with people, and was bestowed with a special gift to be delicate, social, and cheerful with everyone. I never saw him angry, irritable, sad, or depressed: he could find the silver lining to keep pushing toward a good result in any disturbing or dangerous or uncertain situation, and he often helped others to find a new perspective. Kostya represented the good that we all need every day in our professional lives.

Kostya was one of the best clinicians I ever met in my life. He loved helping people, provided treatment, care, comfort, and solace to his patients while asking nothing in return. The volunteers—patients and controls—in our BLT research adored him. I was told that, behind his back, they called a BLT device “the Magic Lamp of Konstantin”. Many expressed their fond memories of the first enrollment in our studies, and often were ready to voluntarily participate again, e.g., in follow-ups and comparisons of different seasons and various options for chronotherapy [6]. 

Shortly after the successful defense of his PhD dissertation (candidate of science) in 1994, he moved to Basel to join the team of Anna Wirz-Justice as her post-doc. Here is a citation from a post by the Centre for Chronobiology [1]:

“His crucial contributions in our laboratory showed that a single dawn simulation at low light intensities phase advances the human circadian core body rhythms. Returning to Novosibirsk, he set up a constant routine lab in his next-door apartment and carried-out some unique and demanding studies: he found that six daily dawn signals were able to forestall the natural delay drift occurring without morning light, and that sleep per se was only a weak Zeitgeber in humans.” [7,8,9]

In Novosibirsk, his work in the constant routine laboratory was linked to other projects of the Institute of Internal Therapy. Then, after the successful defense of his habilitation work (Doctor of Science), he was elected as a vice-director of science at his home research institute in Elzovka, where he worked until his death. He continued to meaningfully contribute to the fields of chronobiology and sleep science and never hesitated to throw himself into the new research projects of this institute. Most recently, he renewed cooperation with the Basel team and was working with Prof. Christian Cajochen “on a joint project, “LightArctic”, about optimizing lighting situations for shift and non-shift workers in the far north of Siberia.” [2]. The remarkable scientific contribution of Dr. Konstantin Danilenko to the fields of chronobiology, chronomedicine, and sleep science was multiplied by his permanent, enthusiastic efforts to help younger generations of researchers to advance their scientific methodologies and the quality of their publications.

Dr. Konstantin Danilenko was a lover of science and spent over 35 last years of his life carrying out scientific research. He was always curious and spoke enthusiastically about the most recent findings in biomedicine. I suppose he would say: “find something you love to do and you’ll never have to work a day in your life”, and that is exactly what he did. Despite being diagnosed with cancer in 2021, Dr. Konstantin Danilenko remained involved in the science and practice of chronobiology until his very last days. He was able to accomplish much before his untimely passing. In particular, together with his colleagues from Novosibirsk and Basel, he proposed a new approach (“hockey stick method”) for the estimation of evening dim light melatonin onset (DLMO) in humans [10]. Most recently, with the help of Dr. Evgeniy Verevkin, he developed a special software for performing such estimations in Microsoft Excel [11]. The latest version of this software was posted on 21 October 2022 on his webpage in ResearchGate [12]. We hope he read the article that was posted on the online site of the Chronobiology International in the middle of December 2022. The authors of this article wrote in the final sentences of their abstract:

“Thanks to its objective nature, the hockey stick method may provide better estimates than the mean of the visual estimations of several raters. These findings suggest that the hockey stick method provides the most reliable estimate of DLMO within the tested methods and should be considered for use in future studies.” [13]

The efforts of Dr. Konstantin Danilenko to explain and educate others on chronobiology and chronomedicine were incredible. Throughout his career, he took an active role in public discussions concerning several chronobiological issues, such as BLT for SAD and daylight savings time. Once, he was included in the group of scientists attending a special session of the Duma to convey their consensus view that the sun must pass a given location’s meridian and reach its highest position in the sky at around 12 o’clock, not an hour later. Moreover, when the right to make the final decision regarding clock times was given to regional authorities, the local dumas of Novosibirsk Oblast and nine other regions decided to make their people healthy, wealthy, and wise by moving the hands of the clock back (i.e., shifting local time further east from Moscow).

Konstantin believed in healthy living and regular exercising. As a favorite hobby, we would often observe him on the football field. The only serious health issue of his that I ever heard about was a severe knee injury during the traditional football match at the ESRS (European Sleep Research Society) congresses in Italy, 2016. As an avid and talented football player, he was passionate about the game and loved to play in competitive tournaments. As the Centre for Chronobiology stated [1]:

“In Basel, Kostya had an extra important function, regularly training every Tuesday evening as a defensive midfield football player for the FC PUK (with our former colleague Kurt Kräuchi), in the team that won the international hospital indoor football tournament in 1998.”

Throughout his career, Konstantin was able to travel extensively in order to attend scientific meetings. He made friends and earned the respect of many of his colleagues. Dr. Konstantin Danilenko served on the Directors’ Board for the Society of Light Treatment and Biological Rhythms (SLTBR) and on the Board of Advisors of the Central Environmental Therapeutics (CET) [2]. We were very fortunate to have had him on the editorial board of our journal [14]. Two publications, an original article [15], and a review [16] were among his contributions. 

The life of Dr. Konstantin Danilenko was well lived, and his work in the fields of chronobiology, chronomedicine, and sleep science will be remembered. We are so grateful for the time we spent with him, and we will greatly miss his expertise, friendship, and humanity.

We hope his family will find the strength to deal with this very painful loss.

## Figures and Tables

**Figure 1:**
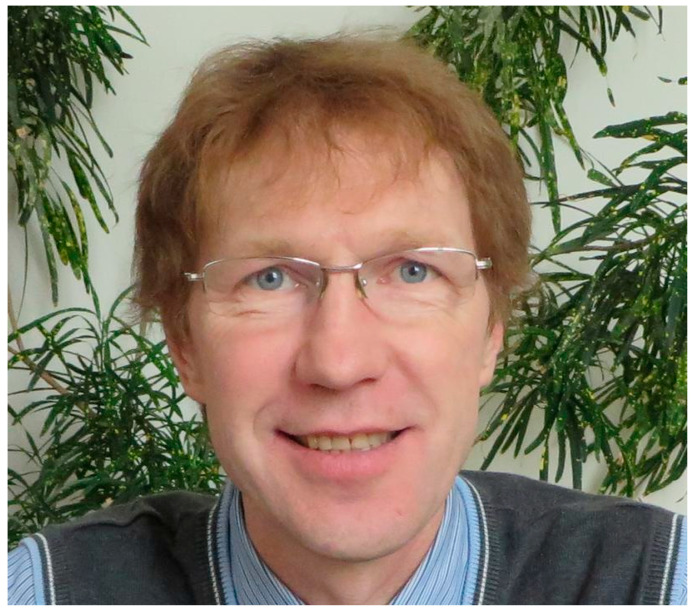
Dr. Konstantin Danilenko.

## Data Availability

Not applicable.

## References

[1] Obituary Konstantin Danilenko. The Centre for Chronobiology. https://www.chronobiology.ch/news/obituary-konstantin-danilenko/.

[2] Cajochen C. In Memory of Konstantin Danilenko (19.03.1962–18.01.2023). https://esrs.eu/news/sleep-science-friday/in-memory-konstantin-danilenko/.

[3] Danilenko K.V., Putilov A.A. (1996). The Importance of Full Summer Remission as a Criterion for the Diagnosis of Seasonal Affective Disorder. Psychopathology.

[4] Danilenko K.V., Putilov A.A. (1993). Diurnal and seasonal variations in cortisol, prolactin, TSH and thyroid hormones in women with and without seasonal affective disorder. J Interdiscipl Cycle Res..

[5] Danilenko K.V., Putilov A.A., Russkikh G.S., Duffy L.K., Ebbesson S.O. (1994). Diurnal and seasonal variations of melatonin and serotonin in women with seasonal affective disorder. Arct. Med. Res..

[6] Danilenko K.V., Putilov A.A. (2005). Melatonin treatment of winter depression following total sleep deprivation: Waking EEG and mood correlates. Neuropsychopharmacology.

[7] Danilenko K.V., Wirz-Justice A., Kräuchi K., Cajochen C., Weber J.M., Fairhurst S., Terman M. (2000). Phase advance after one or three simulated dawns in humans. Chronobiol. Int..

[8] Danilenko K.V., Wirz-Justice A., Kräuchi K., Weber J.M., Terman M. (2000). The human circadian pacemaker can see by the dawn’s early light. J. Biol. Rhythm..

[9] Danilenko K.V., Cajochen C., Wirz-Justice A. (2003). Is sleep per se a zeitgeber in humans?. J. Biol. Rhythm..

[10] Danilenko K.V., Verevkin E.G., Antyufeev V.S., Wirz-Justice A., Cajochen C. (2014). The hockey-stick method to estimate evening dim light melatonin onset (DLMO) in humans. Chronobiol. Int..

[11] Download Hockey-Stick v2.5 Software. Konstantin Danilenko Page in ResearchGate. https://www.researchgate.net/project/Hockey-stick-program-to-approximate-melatonin-onset-and-offset.

[12] Download Hockey-Stick v2.5 Software (21.11.2022). https://cloud.mail.ru/public/1at9/vHwbpU16J.

[13] Glacet R., Reynaud E., Robin-Choteau L., Reix N., Hugueny L., Ruppert E., Geoffroy P.A., Kilic-Huck Ü., Comtet H., Bourgin P. (2022). A comparison of four methods to estimate dim light melatonin onset: A repeatability and agreement study. Chronobiol. Int..

[14] Clocks & Sleep/Editorial Board. https://www.mdpi.com/journal/clockssleep/editors.

[15] Danilenko K.V., Kobelev E., Yarosh S.V., Khazankin G.R., Brack I.V., Miroshnikova P.V., Aftanas L.I. (2020). Effectiveness of Visual vs. Acoustic Closed-Loop Stimulation on EEG Power Density during NREM Sleep in Humans. Clocks Sleep.

[16] Danilenko K.V. (2022). Objective Measures of Immediate "Energizing" Effect of Light: Studies Review and Data Analysis. Clocks Sleep.

